# Effect of the exposure to brominated flame retardants on hyperuricemia using interpretable machine learning algorithms based on the SHAP methodology

**DOI:** 10.1371/journal.pone.0325896

**Published:** 2025-06-26

**Authors:** Yu Cai, Xi-Ru Huang, Sheng-Jia Wang, Ying-Chao Liang, De-Liang Liu, Shu-Fang Chu, Hui-Lin Li

**Affiliations:** 1 The Fourth Clinical Medical College of Guangzhou University of Chinese Medicine, Shenzhen, Guangdong, China; 2 Department of Endocrinology, Shenzhen Traditional Chinese Medicine Hospital, Shenzhen, Guangdong, China; Guangdong Nephrotic Drug Engineering Technology Research Center, Institute of Consun Co. for Chinese Medicine in Kidney Diseases, CHINA

## Abstract

**Background:**

Brominated flame retardants (BFRs) are classified as important endocrine disruptors and persistent organic pollutants; nevertheless, there is no comprehensive investigation to evaluate the association between BFRs and hyperuricemia, and the available studies related to this field are exceptionally scarce.

**Methods:**

For this study, we enrolled 3,812 individuals from NHANES 2005–2016, with nine different types of BFRs serving as the exposure. We conducted advanced machine learning techniques, along with regression analysis to validate our findings from diverse perspectives. Weighted logistic regression were employed to evaluate the association of BFRs for both continuous variables after logarithmic transformation and their quartile subgroups with hyperuricemia.

Restricted cubic spline (RCS) analysis was conducted to identify whether a non-linear relationship exists. Subgroup analysis enabled us to explore potential interactions of research findings across different groups. Weighted quantile sum (WQS) regression was performed to assess collective mixture sum impact, along with contributions of each component. Nine machine-learning models were developed for hyperuricemia prediction, and six discrimination characteristics were applied to select the optimal model. SHapley Additive exPlanations (SHAP) was utilized to interpret the contributions of selected variables for model decision-making capacity.

**Results:**

Several BFRs exhibited noticeable positive correlation with the prevalence of hyperuricemia, including PBDE28 (OR: 1.27, 95% CI: 1.05–1.54, P-value = 0.014), PBDE47 (OR: 1.19, 95% CI: 1.02–1.40, P-value = 0.032), PBDE85 (OR: 1.16, 95% CI: 1.01–1.34, P-value = 0.036), PBDE99 (OR: 1.17, 95% CI: 1.02–1.34, P-value = 0.025), and PBDE154 (OR: 1.16, 95% CI: 1.00–1.34, P-value = 0.050) after fully adjustment. The WQS analysis found that the sum effect of BFRs was positively associated with hyperuricemia, of which PBDE28 (28.70%), PBDE85 (22.10%) and PBDE47 (14.90%) were the top 3 components. XGboost exhibited superior performance across several important metrics. The SHAP analysis revealed that the PBDE85, PBDE28 and PBDE154 exhibited considerable influence, ranking after “BMI≥30”, “Race-Non-Hispanic Black” and “Hypertension-Yes”.

**Conclusions:**

Combining the outcomes, our study identified PBDE28 and PBDE85 as the two major significant contributors to elevated prevalence of hyperuricemia. Other components, such as PBDE154, PBDE47, PBDE99, and PBDE100, emerged as potential pollutants. These pioneering efforts highlighted the previously underrecognized impact on this environmental and public health concern.

## 1. Introduction

Brominated flame retardants (BFRs) are synthetic chemicals that have been widely incorporated into consumer products such as electronics, textiles, and furniture for decades due to their excellent ability to inhibit combustion [1]. However, the persistent nature, high tendency to bio-accumulate, and toxic effects of these substances pose considerable threats to both human health and environmental integrity [2]. Human exposure can occur through multiple pathways, including dietary intake, maternal-infant transmission, product usage, and indoor dust [3]. Studies have found that elevated concentrations of specific BFRs were positively linked to many endocrine disorders, including obesity, hyperlipidemia and diabetes mellitus [4,5]. Furthermore, BFRs exposure has been implicated in a range of adverse health outcomes, including neurodevelopmental issues, thyroid dysfunction, reproductive, liver disorders and chronic kidney disease (CKD) [1,6,7]. BFRs are prone to migrate into water, air, and soil, thereby contributing to widespread environmental contamination [8]. Even though certain BFRs, like Penta- and Octa-BDE, have been phased out in the United States, their lingering presence in consumer items, food sources, and human tissues continues to present notable health concerns [9,10].

Hyperuricemia (HUA) is defined as a metabolic disorder marked by abnormally elevated concentration of uric acid, which is the ultimate product of purine breakdown [11]. According to a recent epidemiological study, the prevalence of hyperuricemia in the U.S. for 2015–2016 was 20.2% in males and 20.0% in females [12]. Although numerous patients exhibit no symptoms, prolonged hyperuricemia serves as a precursor for gout and a risk factor for several diseases conditions, including hypertension, diabetes mellitus, cardiovascular diseases (CVD) and CKD [13,14]. Considering its rising prevalence and connections to multiple chronic conditions, hyperuricemia presents a major public health concern that requires additional research and targeted interventions. Emerging evidence suggested that hyperuricemia was influenced not only by well-established factors such as genetics, high-purine diets, and alcohol consumption but also by environmental pollutants [15–17]. BFRs are classified as important endocrine disruptors and persistent organic pollutants; nevertheless, there is no comprehensive investigation into the potential association between BFRs and hyperuricemia and the available study related to this field are exceptionally scarce. Notably, studies have demonstrated that BDE-209 can induce nephrotoxicity through mechanisms such as renal cell apoptosis, excessive ROS generation, and disturbances in energy metabolism, ultimately compromising kidney function [18]. BDE-47 has been shown to enhance purine metabolism and disrupt glutathione metabolism, leading to oxidative stress and increased uric acid production [19]. Based on these findings, it is essential to elucidate the potential health impacts of BFRs on hyperuricemia.

Machine Learning (ML) employs sophisticated mathematical algorithms to detect and classify patterns in complex, heterogeneous datasets, with the aim of supporting and enhancing decision-making processes [20,21]. Traditional statistical methods for disease identification necessitate specific preconditions for data preparation and a large number of structured data with high-quality distribution [22–24]. Fortunately, ML provides us with the opportunity to identify interactions among high-dimensional data variables [25]. These advanced techniques can process vast and sparse data matrices, thus enabling the analysis of a larger volume of information, facilitating hazard identification and informing health-related decisions [26]. For the interpretation of ML prediction results, SHapley Additive exPlanation (SHAP) provides a clear explanation for each feature’s contribution, serving as a promising and reliable tool for feature selection in medical diagnosis [27].

Our study performed a cross-sectional analysis on the basis of available data from National Health and Nutrition Examination Survey (NHANES) 2005–2016. We employed a variety of statistical methods to thoroughly assess the association between BFRs and hyperuricemia, thereby addressing the current research gap in this area.

## 2. Materials and methods

### 2.1. The participants in the study

The NHANES utilized a multistage and intricately stratified sampling design across the country and has been well recognized as a large-scale updated database. Before inclusion in the database, individuals have already signed a written informed agreement, approved by the National Center for Health Statistics Ethics Review Board (NCHS).

**Fig 1:**
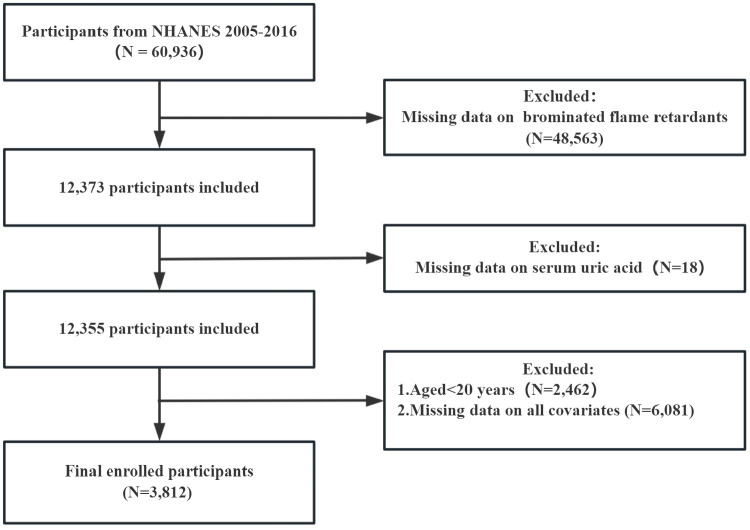
Flowchart of the selection of participants for this study from NHANES 2005–2016.

Our study performed the analysis on the basis of available data from NHANES 2005–2016 and recruited a total of 60,936 participants. First, we excluded participants with missing data of brominated flame retardants (N = 48,563), and with missing data of serum uric acid (N = 18). Second, participants who were <20 years old were excluded (N = 2,462). Finally, individuals lacking information on covariates were also removed from our analysis (N = 6,081). After the above screening, the study included an entire cohort of 3,812 participants (Fig 1).

### 2.2. Assessment of bromine flame retardants

The analysis of twelve different BFRs in serum samples was conducted using an automated liquid/liquid extraction and subsequent sample clean-up. The definitive determination of target analytes was performed utilizing isotope dilution Gas Chromatography/Mass Spectrometry (GC/IDHRMS) [28]. Drawing on earlier studies, nine different types of BFRs detected exceeding 70% were chosen as the exposure to ensure the reliability of our research included PBDE28, PBDE47, PBDE85, PBDE99, PBDE100, PBDE153, PBDE154, PBDE209, and PBB153 [29]. For cases in which the result fell below the detection limit, the variable value depended on dividing the detection limit by the square root of two.

### 2.3. Diagnosis of hyperuricemia

Serum uric acid was measured using a timed endpoint method as part of the routine biochemical analyses, with the data obtained from section of Biochemistry Profile on NHANES website. The diagnosis of hyperuricemia was established when serum concentrations reached≥416 umol/L for males and≥357 umol/L for females.

### 2.4. Covariates

Drawing upon prior investigations, we identified various potential confounding factors, including age, sex, ethnicity, education, poverty-to-income ratio (PIR), smoking status, alcohol consumption, body mass index (BMI), waist circumference (WC), hypertension, hyperlipidemia, diabetes, CKD and CVD. The classifications of smoking status were as follows: 1) Current smoker, those who have smoked exceeding 100 cigarettes in their lifetime and are now involved in smoking behavior either occasionally or daily; 2) Former smoker, who have abstained from smoking until now; 3) Never smoker, those who have smoked less than 100 cigarettes in a lifetime. Our analysis stratified the alcohol consumption into two separate categories determined by whether consume alcohol over 12 times per year. BMI was categorized by the value of 30 kg/m^2^ into two groups. The hypertension was diagnosed when the participants satisfied one or more of the items: 1) Systolic pressure≥140 mmHg; 2) Diastolic pressure≥90 mmHg; 3) Accepting the medication presently; 4) Hypertension that has been told by a healthcare professional. Hyperlipidemia was confirmed when at least one of the following criteria was satisfied: 1) Presently receiving medication for reducing blood lipids; 2) Hypertriglyceridemia (TG ≥ 150 mg/dL); 3) Hypercholesterolemia (TC ≥ 200 mg/dL, LDL-C ≥ 130 mg/dL or HDL < 40mg/dL for men and HDL < 50mg/dL for women). Diabetes mellitus: 1) Fasting glucose≥7 mmol/L; 2) Random plasma glucose≥11.1 mmol/L; 3) OGTT 2-hour plasma glucose≥11.1 mmol/l; 4) The glycohemoglobin > 6.5%; 5) Diabetes that has been told by physicians. The presence of albuminuria (urinary albumin-to-creatinine ratio exceeding 30 mg/g) or a decreased estimated glomerular filtration rate (equal to or more than 60 mL/min/1.73 m^2^) was indicative of chronic kidney disease. Cardiovascular disease referred to a physician’s diagnosis of one or more conditions, including angina, heart attack, congestive heart failure, and coronary artery disease.

### 2.5. Statistical analysis

Our study performed all statistical methods through R software Version 4.4.2, and utilized “sdmvstra”, “sdmvpsu” and “wtint2yr” to generate estimates that were representative of the entire national population for the survey design. Initially, individuals were separated into two groups based on the diagnosis of hyperuricemia, and differences in the demographic characteristics of each group were compared. The Wilcoxon rank-sum test was applied to analyze continuous variables, and the chi-square test for categorical variables. A series of multivariate weighted logistic regression were employed to evaluate the association of BFRs for both continuous variables after logarithmic transformation and their quartile subgroups with hyperuricemia. Model 1 was not adjusted for any variables. Model 2 was adjusted for age, sex, ethnicity, education, PIR, smoking status, alcohol consumption, waist circumference, and BMI. Model 3 was additionally adjusted for hypertension, hyperlipidemia, diabetes, CKD, and CVD. Restricted cubic spline (RCS) analysis was conducted to identify whether a non-linear relationship exists. Subgroup analysis enabled us to explore potential interactions of research findings across different groups. Weighted quantile sum (WQS) regression for nine different BFRs was conducted to assess the collective mixture sum impact, along with contributions of each component by calculating their weights. In the process, we separated the dataset into a 40% for training and 60% for validation. The training set estimated each mixture components’ effect value with 400 bootstrap resamples, along with the combined one, while the testing set was performed to calculate and test the significance for the outcomes. For outcomes of all statistical methods, P-value < 0.05 were considered significant (two‐tailed).

### 2.6. Model development for machine learning

A random split was performed on the dataset for machine learning into training and testing sets with a 7:3 ratio. Multicollinearity among the variables was assessed by the method of variance inflation factor (VIF). Variables with a VIF value exceeding 10 would be discarded from the study [30]. Five-fold cross-validation was employed on the training set to iteratively conduct testing and hyperparameter tuning, ensuring the model’s robustness and determining the optimal configuration. Selected variables were analyzed using nine algorithms, including Decision Tree (DT), Elastic Net (ENET), K-nearest Neighbors (KNN), Light Gradient Boosting Machine (LightGBM), Logistic Regression (LR), Multilayer Perceptron (MLP), Random, Forest (RF), Support Vector Machine (SVM) and Extreme Gradient Boosting (XGBoost). Model accuracy and clinical prediction capability were evaluated using accuracy, the area under the receiver operating characteristic curve (AUC), precision, sensitivity/recall, F1 score and specificity. Calibration curves were used to judge the accuracy of absolute risk prediction. The SHAP method was used to assess the contribution of predictor variables for model interpretability. The method generated a predicted value for each sample, visualized in the SHAP Summary plot, where purple and yellow dots represent high and low feature values, respectively.

## 3. Results

### 3.1. Demographic features of the participants

The demographic features of 3,812 participants were summarized in Table 1, consisting of 731 individuals with hyperuricemia and 3,081 individuals without hyperuricemia. Differences in age, ethnicity, smoking status, BMI, WC, hypertension, hyperlipidemia, diabetes mellitus, CKD, and CVD were statistically significant between the two groups. Additionally, significant differences in BFR concentrations were observed for PBDE28, PBDE47, PBDE85, PBDE99, PBDE100, PBDE154, and PBDE99 (P-value < 0.05).

**Table 1:** Basic characteristics of the study population.

Characteristic	Overall (N = 3812^2^)	Non-hyperuricemia (N = 3081^2^)	Hyperuricemia (N = 731^2^)	P-value
**Age (years)**	48 (17)	47 (17)	53 (18)	**<0.001**
**Age groups**				**<0.001**
≤39	1,271 (35%)	1,101 (36%)	170 (27%)	
40-59	1,243 (38%)	1,040 (39%)	203 (33%)	
≥60	1,298 (27%)	940 (24%)	358 (40%)	
**Sex**				0.7
Male	1,858 (48%)	1,491 (48%)	367 (49%)	
Female	1,954 (52%)	1,590 (52%)	364 (51%)	
**Race**				**<0.001**
Non-Hispanic White	1,720 (70%)	1,390 (70%)	330 (70%)	
Non-Hispanic Black	785 (11%)	584 (10%)	201 (14%)	
Mexican American	590 (7.9%)	510 (8.4%)	80 (5.9%)	
Other Hispanic	383 (5.3%)	332 (5.7%)	51 (3.5%)	
Other/multiracial	334 (5.9%)	265 (5.8%)	69 (6.4%)	
**Education**				0.2
More than high school	2,022 (62%)	1,644 (62%)	378 (59%)	
High school	853 (22%)	670 (22%)	183 (25%)	
Under high school	937 (16%)	767 (16%)	170 (16%)	
**PIR groups**				0.8
<1.0	791 (14%)	644 (14%)	147 (13%)	
≥1, < 3	1,581 (36%)	1,258 (35%)	323 (37%)	
≥3	1,440 (50%)	1,179 (51%)	261 (49%)	
**Smoking Status**				**0.003**
Current smoker	750 (20%)	631 (20%)	119 (17%)	
Former smoker	967 (26%)	741 (24%)	226 (32%)	
Never smoker	2,095 (54%)	1,709 (55%)	386 (50%)	
**Alcohol consumption**				0.2
Yes	2,706 (76%)	2,206 (77%)	500 (74%)	
No	1,106 (24%)	875 (23%)	231 (26%)	
**Waist Circumference (cm)**	99 (16)	97 (15)	108 (16)	**<0.001**
**BMI (kg/m**^**2**^)	29 (7)	28 (6)	32 (8)	**<0.001**
**BMI groups**				**<0.001**
<30	2,390 (64%)	2,085 (69%)	305 (44%)	
≥30	1,422 (36%)	996 (31%)	426 (56%)	
**Hypertension**				**<0.001**
Yes	1,413 (33%)	985 (28%)	428 (54%)	
No	2,399 (67%)	2,096 (72%)	303 (46%)	
**Hyperlipidemia**				**<0.001**
Yes	2,793 (72%)	2,180 (69%)	613 (84%)	
No	1,019 (28%)	901 (31%)	118 (16%)	
**Diabetes**				**<0.001**
Yes	786 (16%)	545 (13%)	241 (26%)	
No	3,026 (84%)	2,536 (87%)	490 (74%)	
**Chronic kidney disease**				**<0.001**
Yes	687 (14%)	429 (11%)	258 (28%)	
No	3,125 (86%)	2,652 (89%)	473 (72%)	
**Cardiovascular disease**				**<0.001**
Yes	300 (6.6%)	189 (5.3%)	111 (12%)	
No	3,512 (93%)	2,892 (95%)	620 (88%)	
**PBDE28 (pg/g)**	8.6 (6.6)	8.4 (6.5)	9.4 (7.3)	**<0.001**
**PBDE47 (pg/g)**	164 (174)	161 (169)	178 (191)	**0.003**
**PBDE85 (pg/g)**	3.7 (4.9)	3.6 (4.7)	4.1 (5.9)	**0.005**
**PBDE99 (pg/g)**	36 (49)	35 (49)	39 (53)	**0.004**
**PBDE100 (pg/g)**	34 (37)	34 (36)	37 (43)	**0.006**
**PBDE153 (pg/g)**	78 (72)	78 (72)	76 (75)	0.2
**PBDE154 (pg/g)**	3.26 (4.06)	3.19 (3.90)	3.57 (4.72)	**0.004**
**PBDE209 (pg/g)**	19 (29)	19 (29)	19 (32)	0.7
**PBB153 (pg/g)**	33 (70)	33 (70)	34 (70)	**<0.001**

^1^N not Missing (unweighted)

^2^Mean (SD); n (unweighted) (%)

### 3.2. Associations between BFRs and hyperuricemia by logistic regression

Table 2 summarized the outcomes of weighted multivariate logistic regression, assessing the impact of nine specific types of BFRs concentrations on the prevalence of hyperuricemia. After controlling for a range of confounders in Model 3, several BFRs still exhibited a noticeable positive correlation with the risk of hyperuricemia, including PBDE28 (OR: 1.27, 95% CI: 1.05–1.54, P-value = 0.014), PBDE47 (OR: 1.19, 95% CI: 1.02–1.40, P-value = 0.032), PBDE85 (OR: 1.16, 95% CI: 1.01–1.34, P-value = 0.036), PBDE99 (OR: 1.17, 95% CI: 1.02–1.34, P-value = 0.025), and PBDE154 (OR: 1.16, 95% CI: 1.00–1.34, P-value = 0.050).

**Table 2:** Association between BFRs and hyperuricemia via weighted multivariate logistic regression.

	Model1			Model2			Model3		
**Characteristic** **(log, pg/g)**	**OR** ^1^	**95% CI** ^1^	**P-value**	**OR** ^1^	**95% CI** ^1^	**P-value**	**OR** ^1^	**95% CI** ^1^	**P-value**
**PBDE28**	1.35	1.15, 1.58	**<0.001**	1.28	1.07, 1.55	**0.009**	1.27	1.05, 1.54	**0.014**
**PBDE47**	1.24	1.07, 1.43	**0.004**	1.20	1.02, 1.41	**0.025**	1.19	1.02, 1.40	**0.032**
**PBDE85**	1.20	1.06, 1.37	**0.005**	1.18	1.02, 1.35	**0.024**	1.16	1.01, 1.34	**0.036**
**PBDE99**	1.19	1.05, 1.35	**0.007**	1.17	1.02, 1.35	**0.024**	1.17	1.02, 1.34	**0.025**
**PBDE100**	1.19	1.04, 1.37	**0.013**	1.18	1.02, 1.37	**0.030**	1.15	0.99, 1.34	0.068
**PBDE153**	0.91	0.78, 1.07	0.246	0.97	0.82, 1.14	0.709	0.93	0.78, 1.10	0.374
**PBDE154**	1.19	1.04, 1.36	**0.013**	1.18	1.01, 1.37	**0.033**	1.16	1.00, 1.34	**0.050**
**PBDE209**	0.96	0.79, 1.16	0.652	1.02	0.83, 1.25	0.874	1.01	0.82, 1.26	0.906
**PBB153**	1.16	1.06, 1.28	**0.002**	1.05	0.92, 1.20	0.473	1.01	0.87, 1.16	0.941

^1^OR = Odds Ratio, CI = Confidence Interval.

Model 1 was adjusted for no variables. Model 2 was adjusted for age, sex, race, education, PIR, smoking status, alcohol consumption, waist circumference, and BMI. Model 3 was further adjusted for hypertension, hyperlipidemia, diabetes, chronic kidney disease, and cardiovascular disease based on Model 2.

Subsequently, the nine BFRs were stratified by quartile for a more detailed examination (Table 3). In the Model 3, a notably increased prevalence of hyperuricemia was mainly established in the following subclasses: the PBDE28 in the 2nd (OR: 1.54, 95% CI: 1.11–2.12, P-value = 0.010), the 3rd (OR: 1.46, 95% CI: 1.00–2.14, P-value = 0.049) and the 4th (OR: 1.70, 95% CI: 1.20–2.42, P-value = 0.004) quartile; the PBDE47 in the 4th (OR: 1.61, 95% CI: 1.11–2.34, P-value = 0.013) quartile; the PBDE85 in the 4th (OR: 1.50, 95% CI: 1.06–2.12, P-value = 0.023) quartile; the PBDE99 in the 4th (OR: 1.46, 95% CI: 1.03–2.06, P-value = 0.033) quartile; the PBDE100 in the 2nd (OR: 1.46, 95% CI: 1.01–2.12, P-value = 0.046), the 3rd (OR: 1.69, 95% CI: 1.17–2.44, P-value = 0.006) and the 4th (OR: 1.44, 95% CI: 1.03–2.02, P-value = 0.032) quartile; the PBDE154 in the 2nd (OR: 1.50, 95% CI: 1.06–2.12, P-value = 0.024), the 3rd (OR: 1.82, 95% CI: 1.20–2.74, P-value = 0.005) and the 4th (OR: 1.60, 95% CI: 1.14–2.25, P-value = 0.007) quartile and the PBDE209 in the 3rd (OR: 1.38, 95% CI: 1.02–1.87, P-value = 0.038) quartile, comparing with those in the reference quartiles.

**Table 3:** Association between brominated flame retardants and hyperuricemia via weighted multivariate logistic regression grouped by quartile.

	Model1			Model2			Model3		
**Characteristic**	**OR** ^1^	**95% CI** ^1^	**P-value**	**OR** ^1^	**95% CI** ^1^	**p-value**	**OR** ^1^	**95% CI** ^1^	**p-value**
**PBDE28 (pg/g)**									
Quartile 1	—	—		—	—		—	—	
Quartile 2	1.54	1.13, 2.08	**0.006**	1.50	1.08, 2.08	**0.016**	1.54	1.11, 2.12	**0.010**
Quartile 3	1.48	1.04, 2.11	**0.030**	1.48	1.02, 2.16	**0.040**	1.46	1.00, 2.14	**0.049**
Quartile 4	1.77	1.33, 2.35	**<0.001**	1.70	1.21, 2.38	**0.003**	1.70	1.20, 2.42	**0.004**
**PBDE47 (pg/g)**									
Quartile 1	—	—		—	—		—	—	
Quartile 2	1.30	0.95, 1.77	0.101	1.31	0.93, 1.84	0.124	1.35	0.94, 1.94	0.099
Quartile 3	1.60	1.07, 2.39	**0.021**	1.54	1.00, 2.37	**0.048**	1.56	0.99, 2.48	0.057
Quartile 4	1.61	1.17, 2.20	**0.004**	1.59	1.11, 2.26	**0.012**	1.61	1.11, 2.34	**0.013**
**PBDE85 (pg/g)**									
Quartile 1	—	—		—	—		—	—	
Quartile 2	1.04	0.71, 1.52	0.858	1.11	0.74, 1.67	0.600	1.10	0.73, 1.65	0.641
Quartile 3	1.49	1.01, 2.18	**0.043**	1.48	0.98, 2.21	0.060	1.51	1.00, 2.30	0.052
Quartile 4	1.51	1.11, 2.04	**0.008**	1.49	1.07, 2.08	**0.020**	1.50	1.06, 2.12	**0.023**
**PBDE99 (pg/g)**									
Quartile 1	—	—		—	—		—	—	
Quartile 2	1.11	0.80, 1.55	0.527	1.15	0.80, 1.66	0.437	1.17	0.79, 1.73	0.422
Quartile 3	1.41	0.95, 2.08	0.085	1.36	0.90, 2.04	0.139	1.42	0.93, 2.17	0.104
Quartile 4	1.46	1.08, 1.97	**0.015**	1.46	1.05, 2.04	**0.024**	1.46	1.03, 2.06	**0.033**
**PBDE100 (pg/g)**									
Quartile 1	—	—		—	—		—	—	
Quartile 2	1.53	1.11, 2.11	**0.010**	1.54	1.09, 2.17	**0.015**	1.46	1.01, 2.12	**0.046**
Quartile 3	1.59	1.15, 2.20	**0.005**	1.60	1.13, 2.26	**0.009**	1.69	1.17, 2.44	**0.006**
Quartile 4	1.50	1.11, 2.02	**0.008**	1.53	1.12, 2.10	**0.009**	1.44	1.03, 2.02	**0.032**
**PBDE153 (pg/g)**									
Quartile 1	—	—		—	—		—	—	
Quartile 2	0.73	0.54, 0.99	**0.040**	0.74	0.54, 1.03	0.074	0.76	0.54, 1.07	0.120
Quartile 3	0.88	0.64, 1.21	0.431	1.01	0.74, 1.37	0.955	1.03	0.75, 1.42	0.842
Quartile 4	0.80	0.60, 1.07	0.129	0.91	0.68, 1.21	0.514	0.85	0.62, 1.15	0.289
**PBDE154 (pg/g)**									
Quartile 1	—	—		—	—		—	—	
Quartile 2	1.37	0.99, 1.89	0.060	1.41	1.01, 1.98	**0.045**	1.50	1.06, 2.12	**0.024**
Quartile 3	1.72	1.19, 2.50	**0.005**	1.70	1.15, 2.54	**0.009**	1.82	1.20, 2.74	**0.005**
Quartile 4	1.57	1.16, 2.12	**0.004**	1.62	1.17, 2.26	**0.005**	1.60	1.14, 2.25	**0.007**
**PBDE209 (pg/g)**									
Quartile 1	—	—		—	—		—	—	
Quartile 2	1.31	0.97, 1.76	0.079	1.38	0.99, 1.92	0.058	1.40	0.99, 1.98	0.058
Quartile 3	1.23	0.93, 1.65	0.151	1.39	1.03, 1.88	**0.033**	1.38	1.02, 1.87	**0.038**
Quartile 4	1.06	0.79, 1.41	0.691	1.19	0.86, 1.65	0.293	1.15	0.82, 1.61	0.408
**PBB153 (pg/g)**									
Quartile 1	—	—		—	—		—	—	
Quartile 2	1.40	0.97, 2.01	0.069	1.13	0.73, 1.73	0.588	1.06	0.67, 1.67	0.812
Quartile 3	1.55	1.14, 2.10	**0.006**	1.09	0.70, 1.68	0.709	0.98	0.62, 1.56	0.931
Quartile 4	1.69	1.20, 2.38	**0.003**	1.16	0.70, 1.92	0.557	0.96	0.56, 1.65	0.874

^1^ OR = Odds Ratio, CI = Confidence Interval

Model 1 was adjusted for no variables. Model 2 was adjusted for age, sex, race, education, PIR, smoking status, alcohol consumption, waist circumference, and BMI. Model 3 was further adjusted for hypertension, hyperlipidemia, diabetes, chronic kidney disease, and cardiovascular disease based on Model 2.

**Fig 2:**
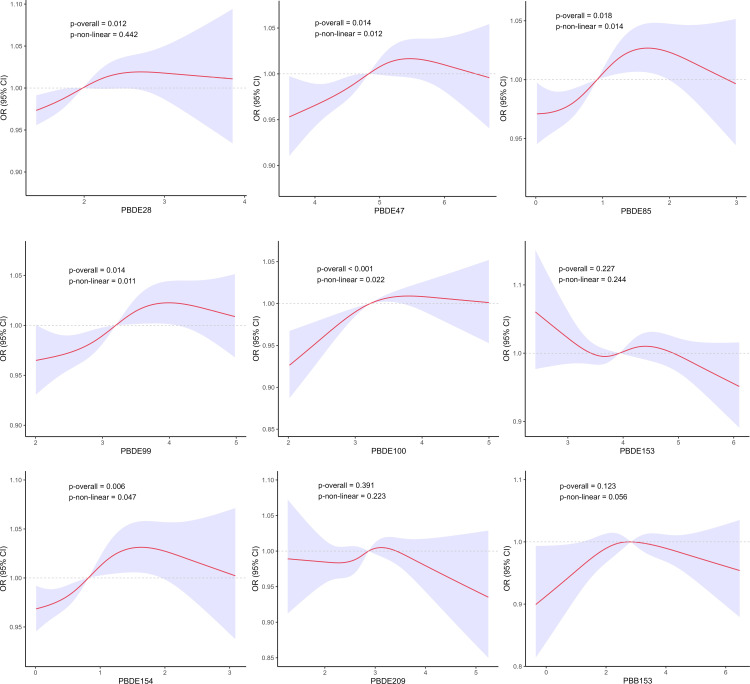
RCS analysis of nine BFRs with hyperuricemia.

As depicted in Fig 2, the RCS analysis revealed that PBDE47 (P for non-linearity = 0.012), PBDE85 (P for non-linearity = 0.014), PBDE99 (P for non-linearity = 0.011), PBDE100 (P for non-linearity = 0.022) and PBDE154 (P for non-linearity = 0.047) exhibited nonlinear interactions with hyperuricemia.

### 3.3. Subgroups analysis

A subgroup analysis was carried out to investigate potential links between statistically significant BFRs identified by logistic regression and hyperuricemia across various subgroups defined by age, sex, race, education, PIR, BMI, cigarette use, drinking status, diabetes, hypertension, CKD, hyperlipidemia and CVD. The findings of the research, as depicted in Fig S1-S6, demonstrated that the association between specific BFRs and hyperuricemia remained consistent across the majority of the subgroups examined. Significant interactive effects were detected within the Hypertension subgroup in PBDE28 (P for interaction = 0.044), Hyperlipidemia subgroup in PBDE47 (P for interaction = 0.026), Hyperlipidemia subgroup in PBDE85 (P for interaction = 0.023) and Hyperlipidemia subgroup in PBDE99 (P for interaction = 0.034).

### 3.4. Combined mixture sum effect of BFRs on hyperuricemia by weighted quantile sum (WQS) regression

**Fig 3:**
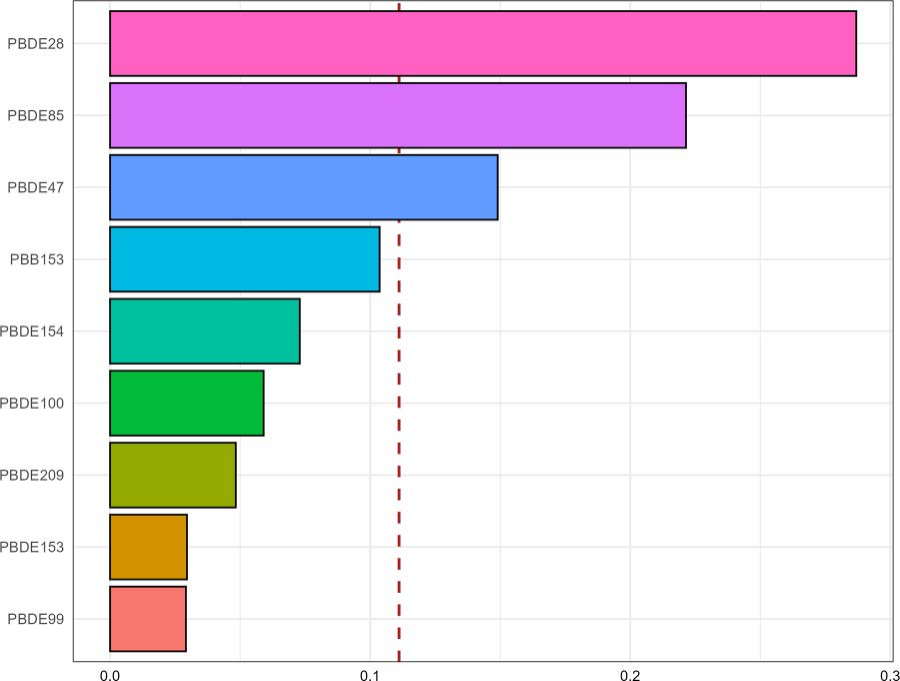
Contributions of each component in WQS regression for BFRs on hyperuricemia with full covariate adjustment.

The WQS model (Fig 3) demonstrated their mixed effect was related to a increased prevalence of hyperuricemia (OR: 1.19, 95% CI: 1.04–1.37, P-value = 0.012). In terms of specific effect estimates of BFRs, PBDE28 (28.70%), PBDE85 (22.10%) and PBDE47 (14.90%) were the top 3 components, followed by PBB153 (10.40%), PBB154(7.29%), PBDE100(5.90%), PBDE209(4.83%), PBDE153(2.96%), and PBDE99(2.92%).

### 3.5. Nine machine learning models and feature importance visualization via SHAP methodology

**Fig 4:**
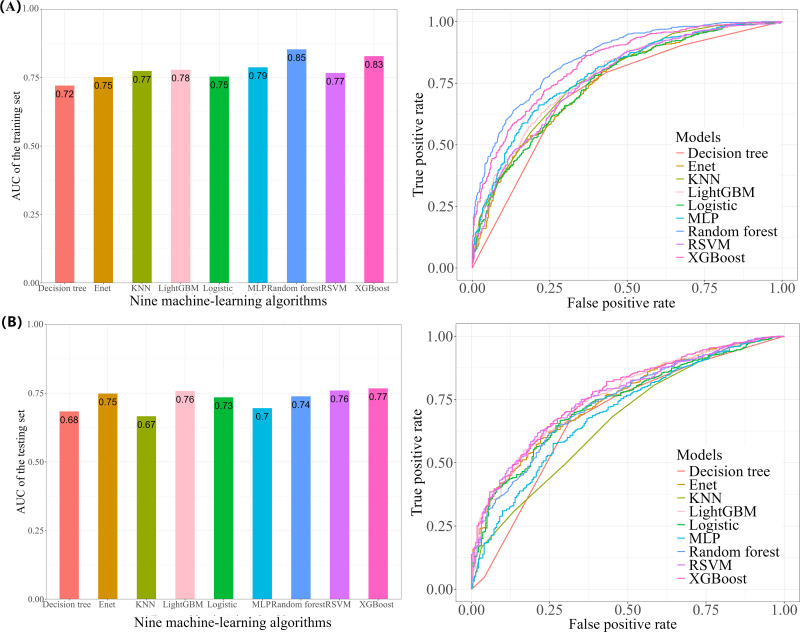
The ROC curves of nine machine-learning models. (A) Training set. (B) Testing set.

**Fig 5:**
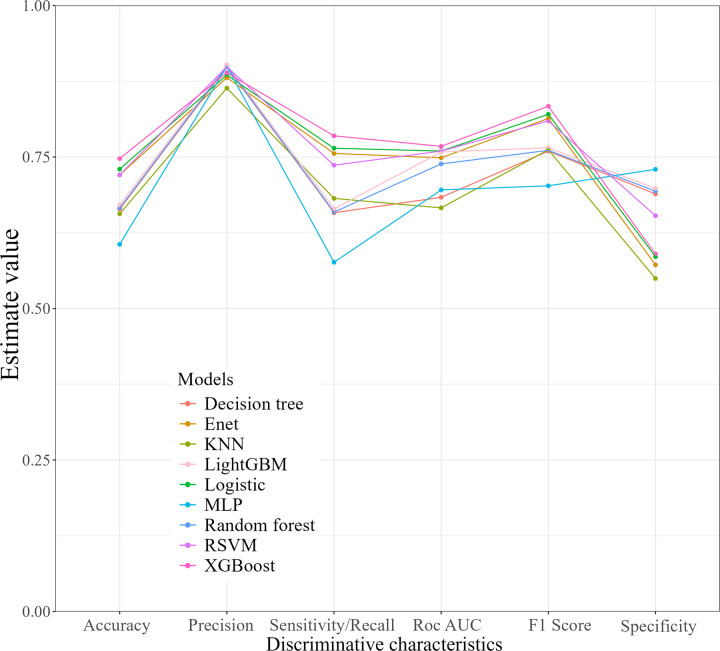
Discriminative characteristics of nine machine-learning models.

**Fig 6:**
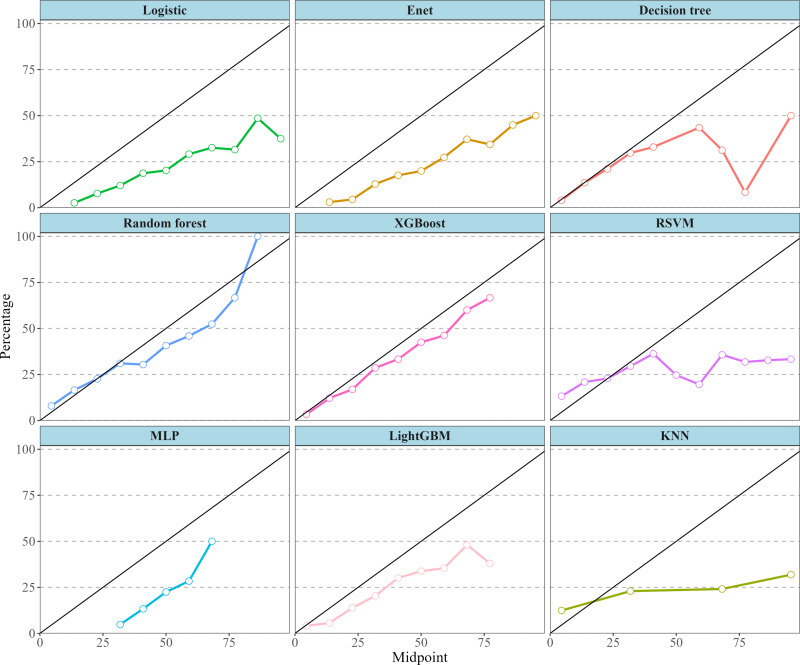
Calibration curve of nine machine-learning models.

Before the construction of the machine learning model, VIF was utilized to examine the multicollinearity. The VIF values for PBDE28 (2.7), PBDE47 (6.6), PBDE85 (4.2), PBDE99 (4.7), PBDE100 (4.9), PBDE153 (1.4), PBDE154 (5.1), PBDE209 (1.1) and PBB153(1.2) were all below the commonly accepted threshold of 10 [30]. The ROC curves for both the training set (Fig 4A) and the testing set (Fig 4B) of nine machine learning models were provided. Table 4 and Fig 5 presented detailed discriminative characteristics for nine machine-learning algorithms. From the results, XGboost exhibited superior performance across several important metrics with the highest Accuracy (0.75), Sensitivity/Recall (0.78), AUC (0.77), and F1 score (0.83), in addition to a remarkable Precision (0.89). Additionally, the calibration curve (Fig 6) of the XGboost closely matched the reference line, indicating its exceptional predictive accuracy. As illustrated in the Fig S7, the fluctuations in the validation results across each fold of XGboost was less than 10%, indicating the model’s reliability and robustness.

**Table 4:** Detailed discriminative characteristics of ROC curve for nine machine-learning algorithms.

Model	Accuracy	Precision	Sensitivity/Recall	AUC	F1 score	Specificity
**Decision tree**	0.66	0.90	0.66	0.68	0.76	0.69
**Enet**	0.72	0.88	0.76	0.75	0.81	0.57
**KNN**	0.66	0.86	0.68	0.67	0.76	0.55
**LightGBM**	0.67	0.90	0.66	0.76	0.77	0.70
**Logistic**	0.73	0.89	0.76	0.76	0.82	0.59
**MLP**	0.61	0.90	0.58	0.70	0.70	0.73
**Random forest**	0.67	0.90	0.66	0.74	0.76	0.69
**RSVM**	0.72	0.90	0.74	0.76	0.81	0.65
**XGBoost**	**0.75**	0.89	**0.78**	**0.77**	**0.83**	0.59

**Fig 7:**
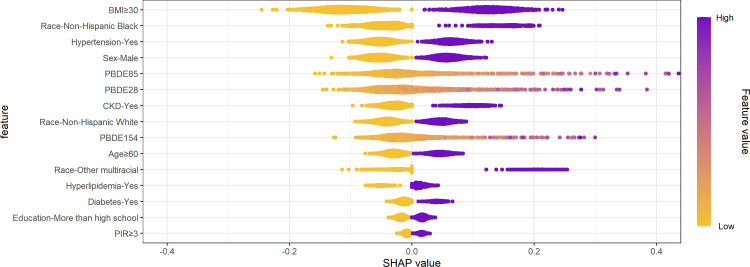
SHAP Summary plot based on the XGboost.

Subsequently, the distribution of SHAP values on the basis of XGboost (Fig 7) provided insight into the contributions of various features to the predictions. The color scheme of purple for high values and yellow for low values effectively represented the varying degrees of feature importance. Notably, the variable “BMI≥30” exhibited the most substantial influence on the predicted outcome, with higher SHAP values on the right side corresponding to a positive relationship with the outcome (hyperuricemia). Among the components of BFRs, PBDE85, PBDE28 and PBDE154 exhibited considerable influence, ranking after “Race-Non-Hispanic Black” and “Hypertension-Yes”.

## 4. Discussion

To the best of our knowledge, our study represents the pioneering attempt to systematically evaluate the effects of BFRs on hyperuricemia using NHANES 2005–2016 data, and no study has previously reported this positive association at the epidemiological level. Moreover, this study also constitutes the first effort to develop nine machine learning models as a promising tool for feature selection related to BFRs exposure in medical prediction, utilizing the explainable SHAP methodology. Surprisingly, the only existing comprehensive study, which examined the effects of various persistent toxic substances (PTSs) on hyperuricemia using a sample of 950 Spanish participants, reported an unforeseen inverse correlation between PBDEs and hyperuricemia [31]. Researchers suggested that the similar physicochemical properties of various PTSs might cause false-positive findings, and the effect of participants from different ethnicity and location in the sample selection also required further investigation.

Our study has provided novel and multiple evidence regarding the possible effects of BFRs on hyperuricemia by conducting advanced machine learning techniques, along with regression analysis and WQS regression to validate our findings from diverse perspectives. In the initial analysis, multiple weighted logistic regression models have demonstrated that some BFRs, namely PBDE28, PBDE47, PBDE85, PBDE99, and PBDE154, were significantly correlated with a higher prevalence of hyperuricemia after fully adjusting for covariates. Stratification by quartile further confirmed these significant associations, while PBDE100 emerged as an additional pollutant with a possible correlation. The RCS analysis indicated the nonlinear interactions between some specific BFRs including PBDE47, PBDE85, PBDE99, PBDE100 and PBDE154 with hyperuricemia. This interaction may potentially account for the marginal significance of PBDE154 (P-value = 0.050). Moreover, we employed the WQS regression model to evaluate mixed exposures, given its superior ability to stimulate intricate exposure scenarios in the real world including the interactions among environmental chemicals [32]. Compared to analyzing individual pollutant separately, this method exhibits enhanced robustness when addressing multicollinearity within flame retardant mixtures, facilitating the precise identification of key components. The findings indicated that the combined effect was linked to an elevated prevalence of hyperuricemia, and PBDE28, PBDE85, and PBDE47 were recognized as the the top three major contributors. Furthermore, ML employs sophisticated mathematical algorithms to detect and classify patterns in complex, heterogeneous datasets, with the aim of supporting and enhancing decision-making processes [20,21]. SHAP methodology incorporates the individual effects of various features as well as the effects arising from their interactions [27]. Based on SHAP summary plots, we obtained the potential BFRs candidates that might present innovative research ideas and draw reasoned conclusions for investigation. We performed a comprehensive evaluation of the predictive capabilities of machine learning models and six discrimination characteristics were applied to select the model with optimal performance. Among the nine machine-learning algorithms, the XGboost exhibited superior performance across several important metrics with the highest Accuracy (0.75), Sensitivity/Recall (0.78), AUC (0.77), and F1 score (0.83), in addition to a remarkable Precision (0.89). The calibration curve of the XGboost closely matched the reference line, indicating its exceptional predictive accuracy and stability. The SHAP interpretability analysis revealed that the components of BFRs, namely PBDE85, PBDE28 and PBDE154 exhibited considerable influence, ranking after “BMI≥30”, “Race-Non-Hispanic Black” and “Hypertension-Yes”. This finding aligns with previous in vitro experimental evidence, which demonstrated that certain BFRs could promote uric acid production [19], highlighting their potential to induce hyperuricemia.

Although the specific biological mechanisms between BFRs and hyperuricemia are limited and not fully elucidated, we have collated the existing evidence and proposed several potential pathways. The production of uric acid is under the regulatory control of xanthine oxidoreductase (XOR), the pivotal enzyme that determines the rate of purine catabolism [33]. XOR plays a critical role in catalyzing the sequential conversion of hypoxanthine to xanthine and then to uric acid, constituting the final two steps of purine catabolism [34]. An experiment utilizing mouse preadipocyte cells has demonstrated that BDE47 significantly upregulated purine metabolism in adipocytes by augmenting the xanthine oxidase (XOR) expression, consequently promoting uric acid generation and exacerbating oxidative stress [19]. In addition, the multifaceted association between oxidative stress and serum uric acid concentration has been a persistent focus of scientific research over the past several decades [35]. Existing studies have found that higher oxidative stress are associated with an elevated prevalence of hyperuricemia [36]. Investigation has confirmed that exposure to BDE-47 induced a significant elevation in reactive oxygen species (ROS) levels and differential expression of oxidative stress-related genes in mouse macrophages [37]. The upregulation of oxidant-producing enzymes, representing elevated oxidative stress, also enhanced the activity of xanthine oxidase (XO) enzymes [38].Another research conducted on human embryonic kidney cells revealed that BDE-47 induced cell apoptosis and excessive generation of reactive oxygen species [39].The findings of another animal experiment indicated that relatively low doses of BDE-209 could adversely affect renal function, with oxidative stress identified as a primary mechanism in its nephrotoxicity [18]. The majority of uric acid is naturally excreted by the kidneys, and approximately 90% of individuals with hyperuricemia have insufficient uric acid excretion, linked to reduced glomerular filtration and impaired tubular secretion [11]. A comprehensive clinical study has demonstrated that exposure to certain BFRs was significantly associated with changes in renal function indicators, heightening the potential risk of CKD. Earlier investigations of rat models exposed to BDE-99 identified phagolysosomes in the kidneys, causing kidney dysfunction and an elevation in catalase levels [40].Moreover, research has shown that BFRs accumulating in the kidneys of cats result in necrosis and the shedding of renal tubular epithelial cells [41]. Based on the above studies, we believe that the positive association between BFRs and hyperuricemia observed in our study is reasonable and reliable; however, the specific mechanisms necessitate further investigation for elucidation.

This study possesses several strengths. Our study represents the pioneering attempt to systematically evaluate the effects of BFRs on hyperuricemia. This emphasizes the need for more efforts and research to focus on this environmental health concern. Furthermore, we conducted advanced machine learning techniques, along with regression analysis to validate our findings from diverse perspectives. Compared with the previous non-specific targeted studies, we have reproposed well-substantiated conclusions by a variety of statistical methods and a larger database, addressing the existing research gap in this field. Additionally, appropriate sampling weights were considered to reduce potential oversampling bias and enhance the generalizability of the conclusion to the U.S. population. Despite its strengths, our study has several limitations. Our study examined nine types of BFRs; however, numerous emerging alternatives to BFRs such as PBT and BTBPE, were not incorporated due to the limited data available in NHANES. The findings from NHANES are primarily applicable to the U.S. population; therefore, the current results cannot be fully extrapolated to different ethnic population for example as those in Asia. Despite our efforts to control for relevant confounders in the current model, the possibility of unaccounted covariates cannot be entirely excluded. As a cross-sectional study, further prospective longitudinal cohort studies and animal experiments are required to validate the specific mechanisms and causal relationships between BFRs and hyperuricemia.

## 5. Conclusion

Combining the outcomes, our study identified PBDE28 and PBDE85 as the two major significant contributors to elevated prevalence of hyperuricemia. Other components, such as PBDE154, PBDE47, PBDE99, and PBDE100, emerged as potential pollutants. These pioneering efforts highlighted the previously underrecognized impact on this environmental and public health concern.

## Supporting information

S1 Fig The subgroup analysis of PBDE28 with hyperuricemia. (PNG)

S2 Fig The subgroup analysis of PBDE47 with hyperuricemia. (PNG)

S3 Fig The subgroup analysis of PBDE85 with hyperuricemia. (PNG)

S4 Fig The subgroup analysis of PBDE99 with hyperuricemia. (PNG)

S5 Fig The subgroup analysis of PBDE100 with hyperuricemia. (PNG)

S6 Fig The subgroup analysis of PBDE154 with hyperuricemia. (PNG)

S7 Fig The average ROC values from the five-fold cross-validation of nine ML models. (PNG)
