# Supplementary figures and images for: Effect of the exposure to brominated flame retardants on hyperuricemia using interpretable machine learning algorithms based on the SHAP methodology
Source: PLoS One. 2025 Jun 26;20(6):e0325896. doi: 10.1371/journal.pone.0325896 (PMC12200863; doi:10.1371/journal.pone.0325896)

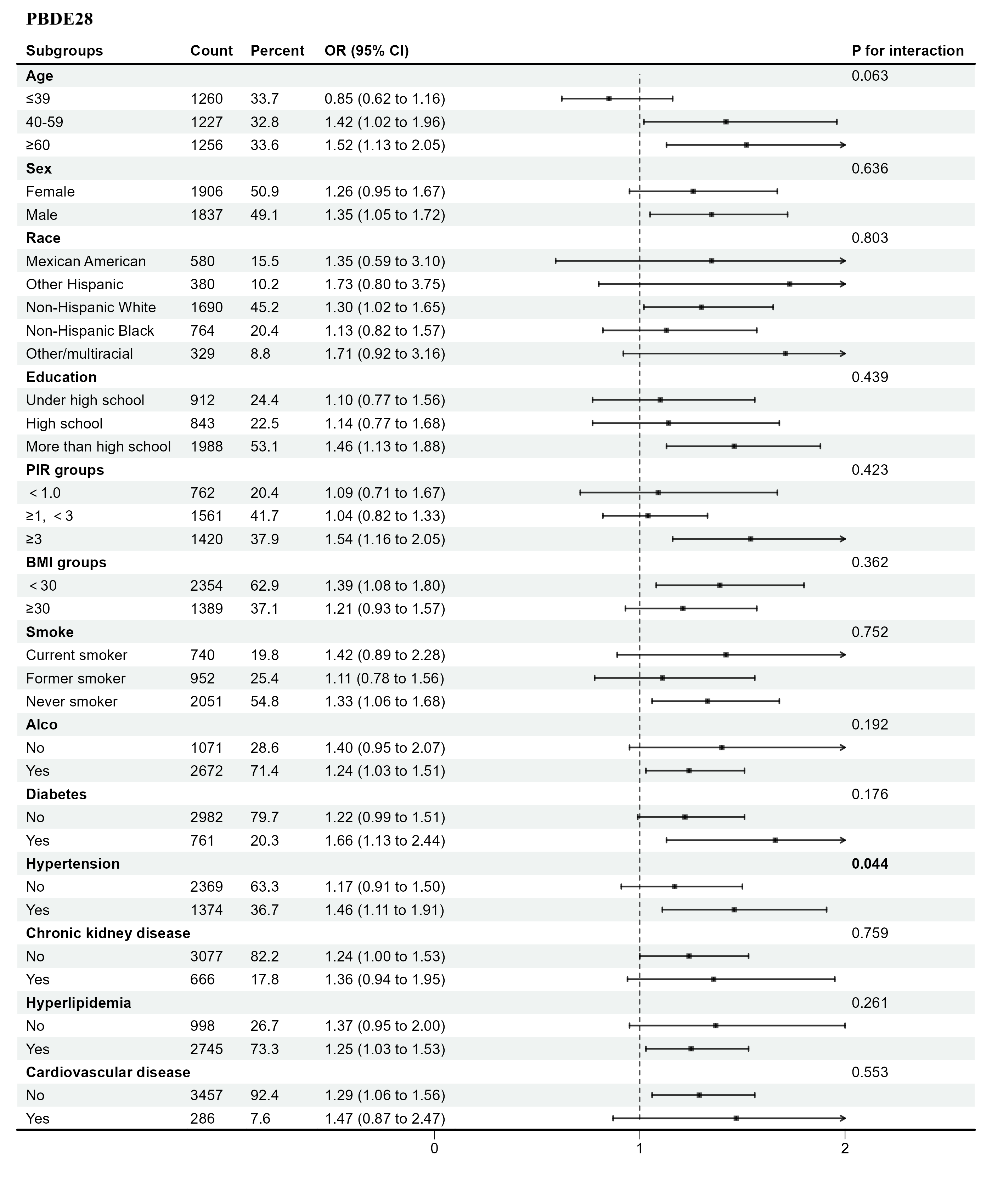

Supplement: S1 Fig — (PNG) [file pone.0325896.s001.png]

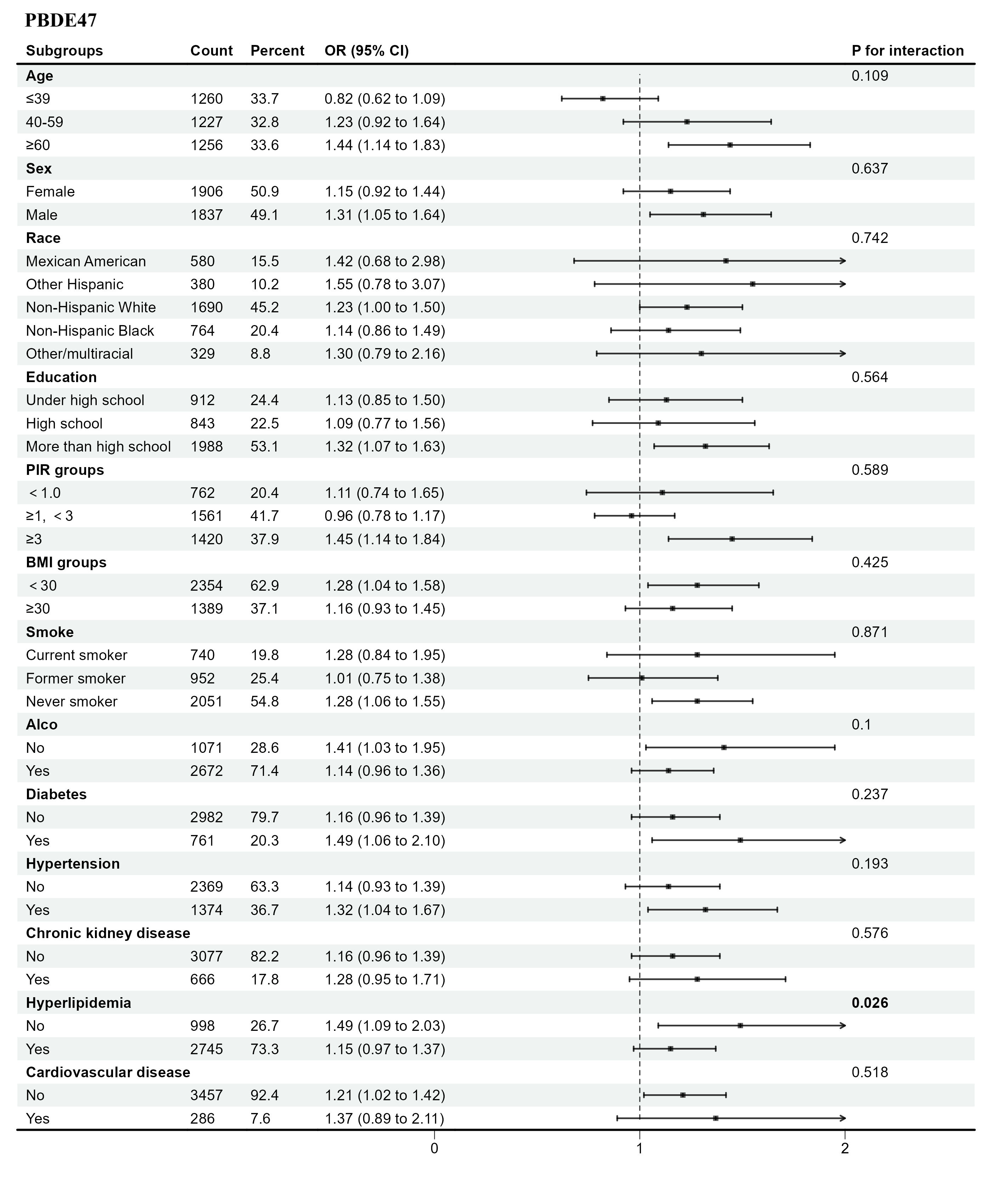

Supplement: S2 Fig — (PNG) [file pone.0325896.s002.png]

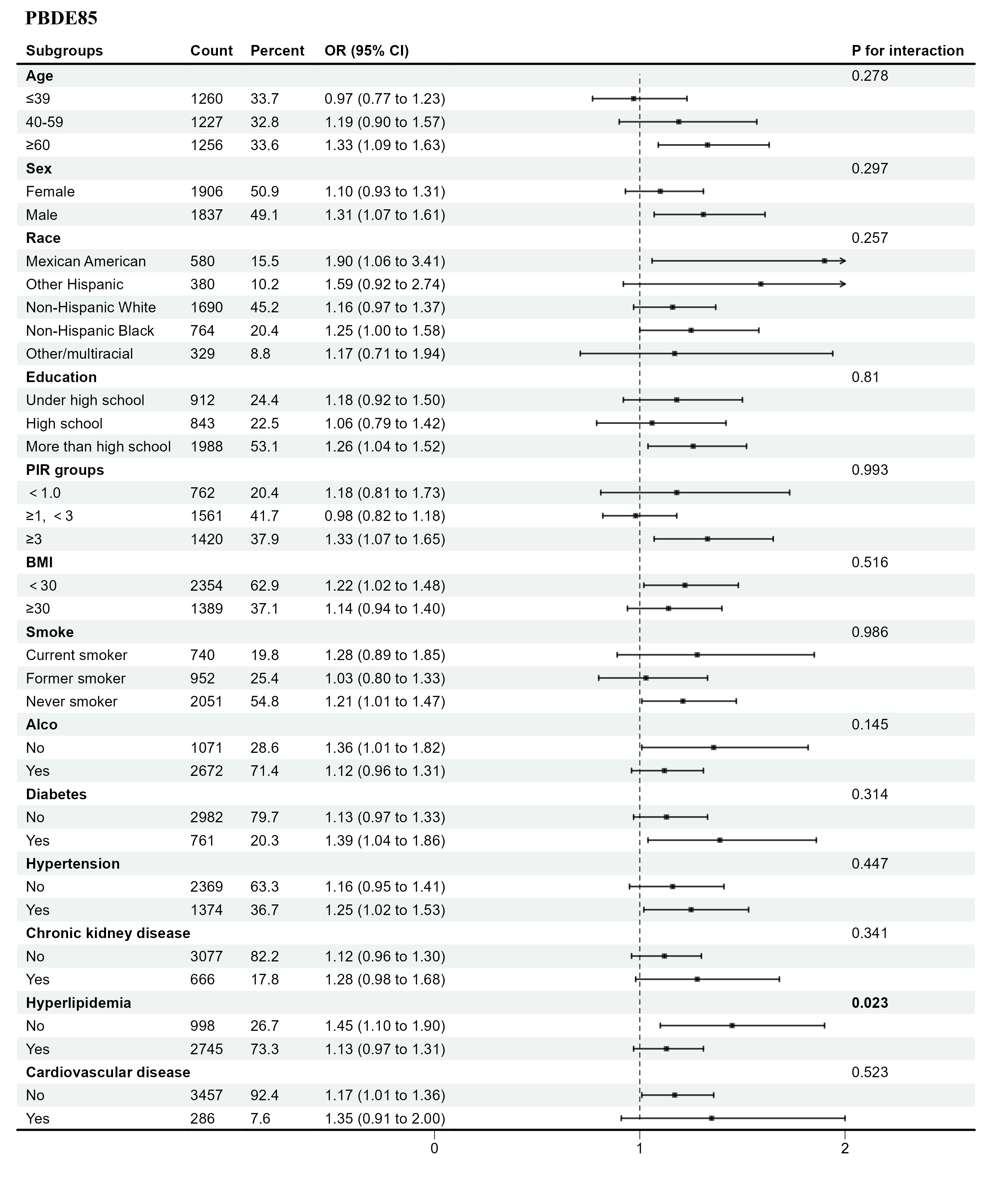

Supplement: S3 Fig — (PNG) [file pone.0325896.s003.png]

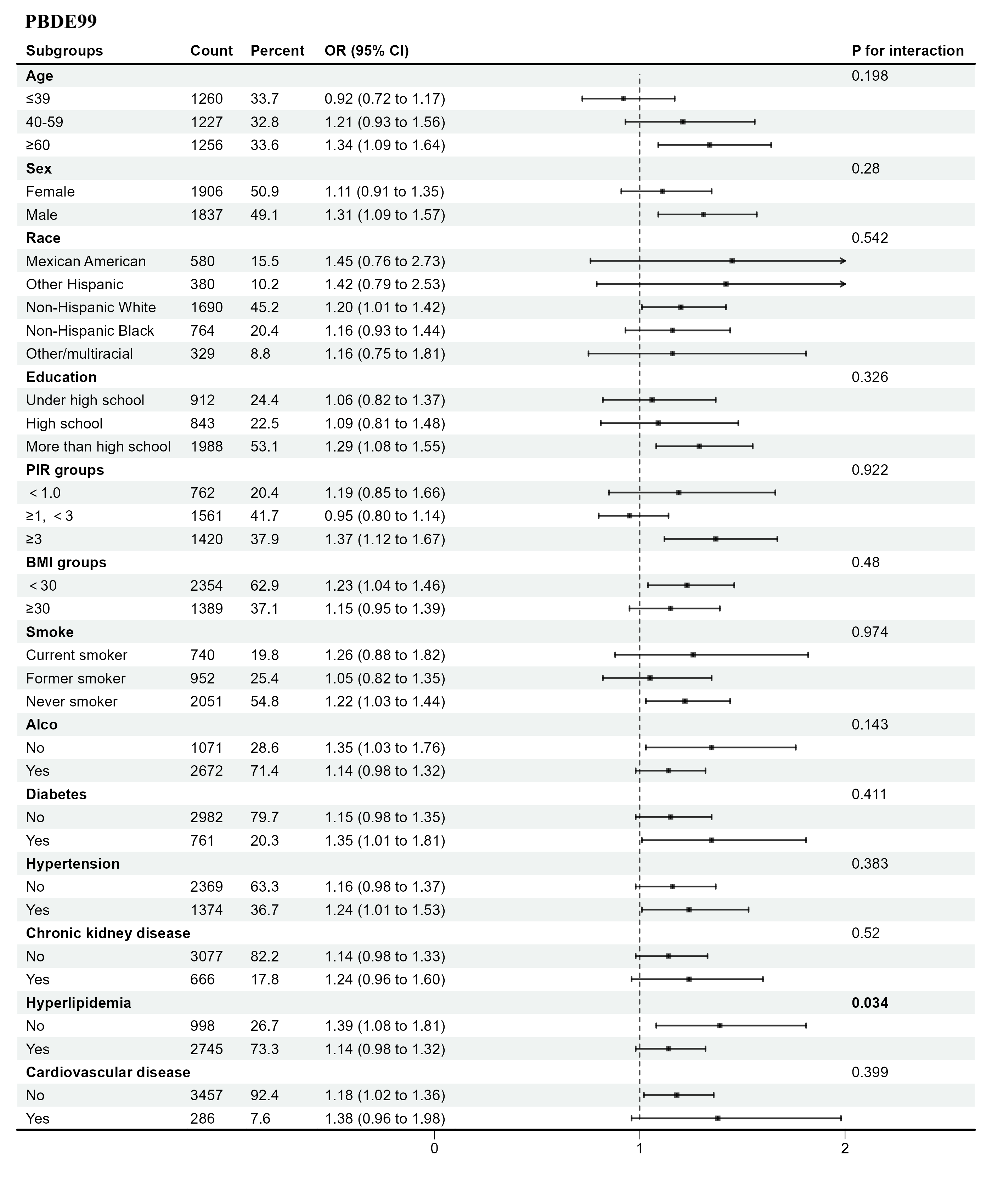

Supplement: S4 Fig — (PNG) [file pone.0325896.s004.png]

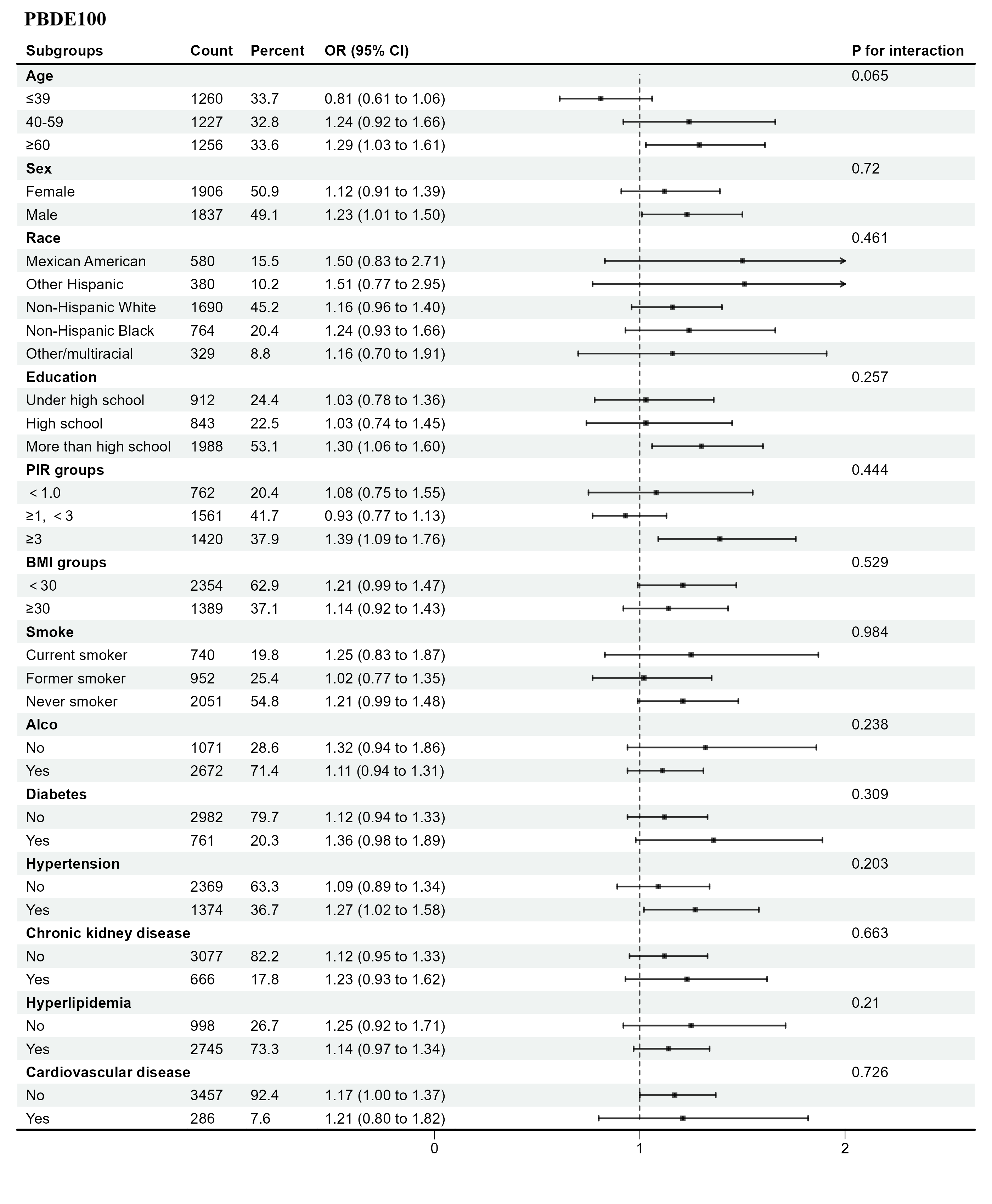

Supplement: S5 Fig — (PNG) [file pone.0325896.s005.png]

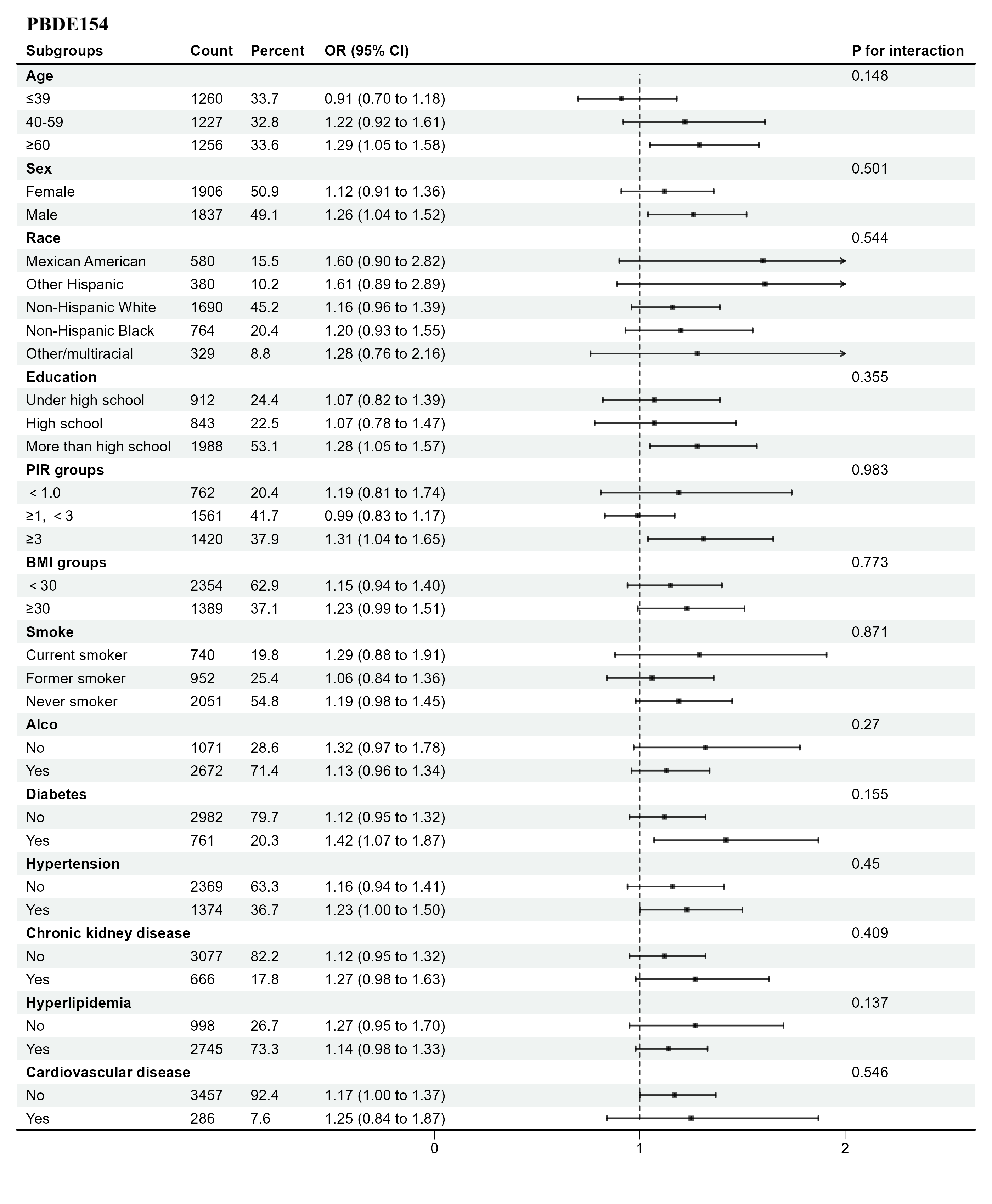

Supplement: S6 Fig — (PNG) [file pone.0325896.s006.png]

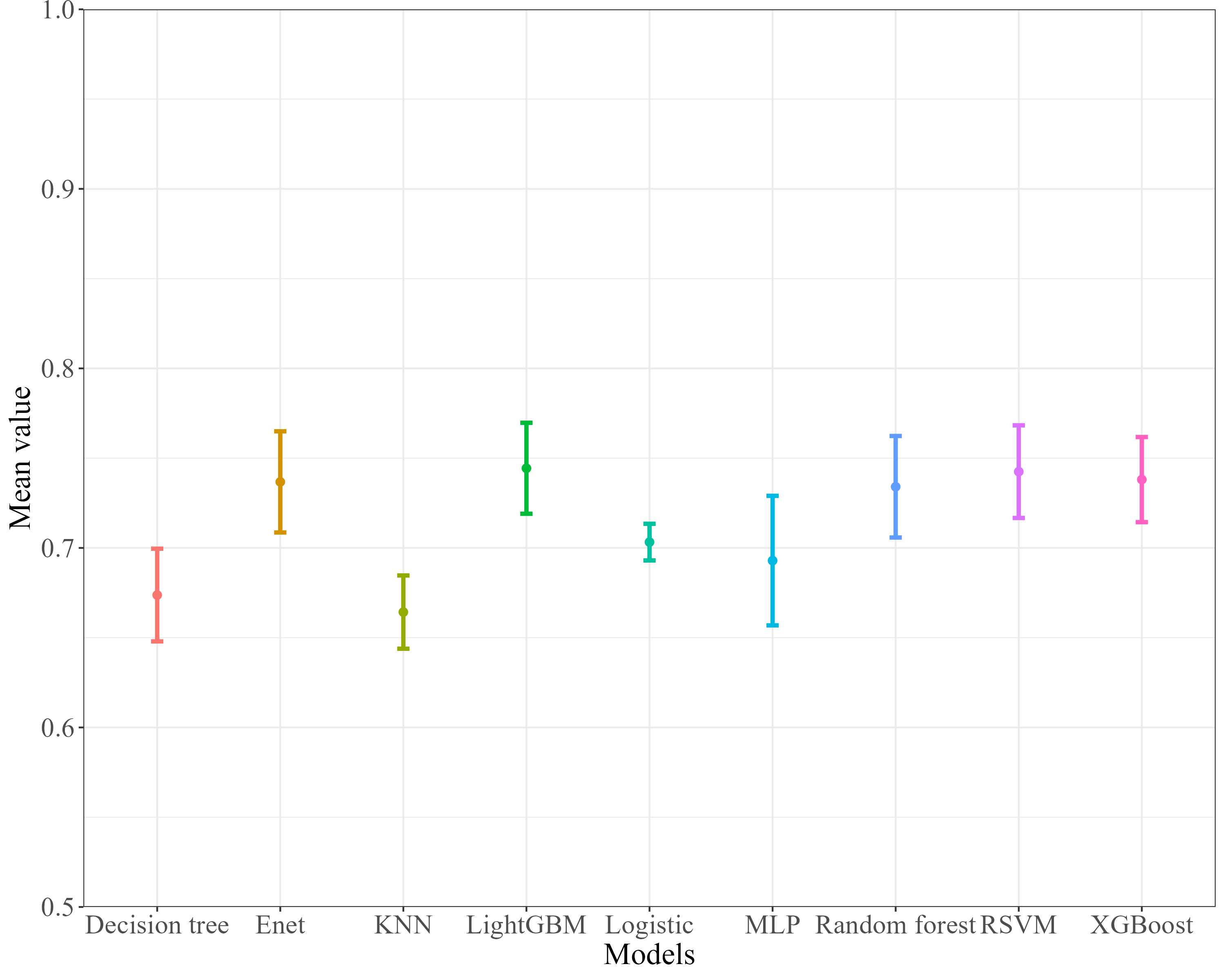

Supplement: S7 Fig — (PNG) [file pone.0325896.s007.png]
